# Case report: risk of skin necrosis related to injectable vancomycin in critically ill newborn infants

**DOI:** 10.1186/s12887-021-02824-8

**Published:** 2021-08-13

**Authors:** Sixtine Gilliot, Mohamed Riadh Boukhris, Morgane Masse, Laurent Storme, Bertrand Décaudin, Pascal Odou, Kevin Le Duc

**Affiliations:** 1 Univ. Lille, CHU Lille, ULR 7365 - GRITA - Groupe de Recherche sur les formes Injectables et les Technologies Associées, F-59000 Lille, France; 2 Institut de Pharmacie, CHU Lille, 59000 Lille, France; 3 Univ. Lille, CHU Lille, ULR 2694– METRICS: Evaluation des technologies de santé et des pratiques médicales, F-59000 Lille, France; 4 Department of Neonatology, Jeanne de Flandre Hospital, University Hospital of Lille, F-59000 Lille, France; 5 Institute/University/Hospital : CHU Lille, Hôpital Jeanne de Flandre, Pôle Femme, mère et Nouveau-né, Avenue Eugène Avinée, 59120 Lille, Loos France

**Keywords:** Vancomycin, Adverse drug event, Neonatal intensive care, Neonatal sepsis

## Abstract

**Background:**

Vancomycin is commonly used as part of empiric antibiotic therapy in the preterm infants who develop signs and symptoms of infection. Although skin necrosis has been noted to occur following injection of vancomycin into a peripheral vein in an adult patient, this complication has not been previously described in a preterm infant.

**Case presentation:**

We report the case of a very low birthweight male infant born at 30 weeks gestational age who developed skin necrosis, most likely as a complication of vancomycin administration via a peripheral venous catheter. The immature skin and endothelial cells of this preterm infant may have increased the risk of drugs related venous and skin toxicity. In this case, assumption of a cumulative toxicity with other drugs administered concomitantly via the same catheter can’t be excluded.

**Conclusions:**

To prevent the risk of skin damage, we advocate that in newborn infants, the administration of vancomycin should be limited to a concentration of < 2.5 mg/mL via a peripheral intravenous catheter if a central venous catheter is not available.

## Background

Staphyloccocus aureus and coagulase negative staphylococci are among the most common pathogens leading to late-onset sepsis in neonates [1]. As a result, vancomycin continues to be commonly used to provide empiric gram-positive coverage when invasive bacterial infection is suspected in a critically ill neonate [2]. Given the widespread use of vancomycin in this vulnerable population, it is important for the clinician to be aware of both common and rare side effects associated with this medication.

The most common cutaneous adverse effect related to the infusion of vancomycin is the red man syndrome which consists of a pruritic erythematous rash on the face, the neck and upper torso, associated with hypotension, fever, chills, evolving towards angio-oedema in the most serious cases, occurring after the rapid infusion of the first administration of the drug. These symptoms occur due to an anaphylactoid reaction where histamine is released from mast cells and basophils independent of pre-formed IgE or complement.. To prevent its occurrence, international guidelines have warned that vancomycin should be administered diluted and infused over a period of at least 60 min or at a rate of 10 to 15 mg/min (≥1 h per 1000 mg) to minimize infusion-related adverse events [3].

A less known adverse event related to the infusion of vancomycin in adults has been reported in the literature: skin necrosis [4]. We aim to promote awareness concerning the occurrence of a skin necrosis related to a 5 mg/mL concentration of vancomycin infusion in a preterm infant.

## Case presentation

A male infant born at 30 weeks’ gestational age with a birthweight of 1380 g was admitted to the neonatal intensive care unit (NICU) at Lille University Hospital in a context of induced prematurity for an antenatal diagnosed Ebstein anomaly with functional pulmonary atresia needing treatment with prostin until surgery. Invasive ventilation for pulmonary hypoplasia lasted 5 days. He displayed adrenal insufficiency requiring corticosteroid supplementation. Concerning his feeding status, he was given individualized parenteral nutrition from birth, and blood glucose management was performed with continuous insulin therapy.

The infant’s hospital course was complicated by suspected sepsis requiring the intravenous antibiotic therapy. Intravenous vancomycin therapy started on the 6th day of life based on clinical symptoms of bacteremia.

On the 8th day of life, the deterioration of his hemodynamic status required the use of vasopressor (dopamine 2.5 μg/kg/min).

The day he developed a skin lesion, he weighed 1245 g. The ventilatory support was performed with Biphasic Positive air pressure (BiPAP, 30% fraction of inspired oxygen). He had lactic acidosis (pH = 6.99, blood lactate level = (3.35 mmol/L). C-reactive protein was negative.

**Fig. 1:**
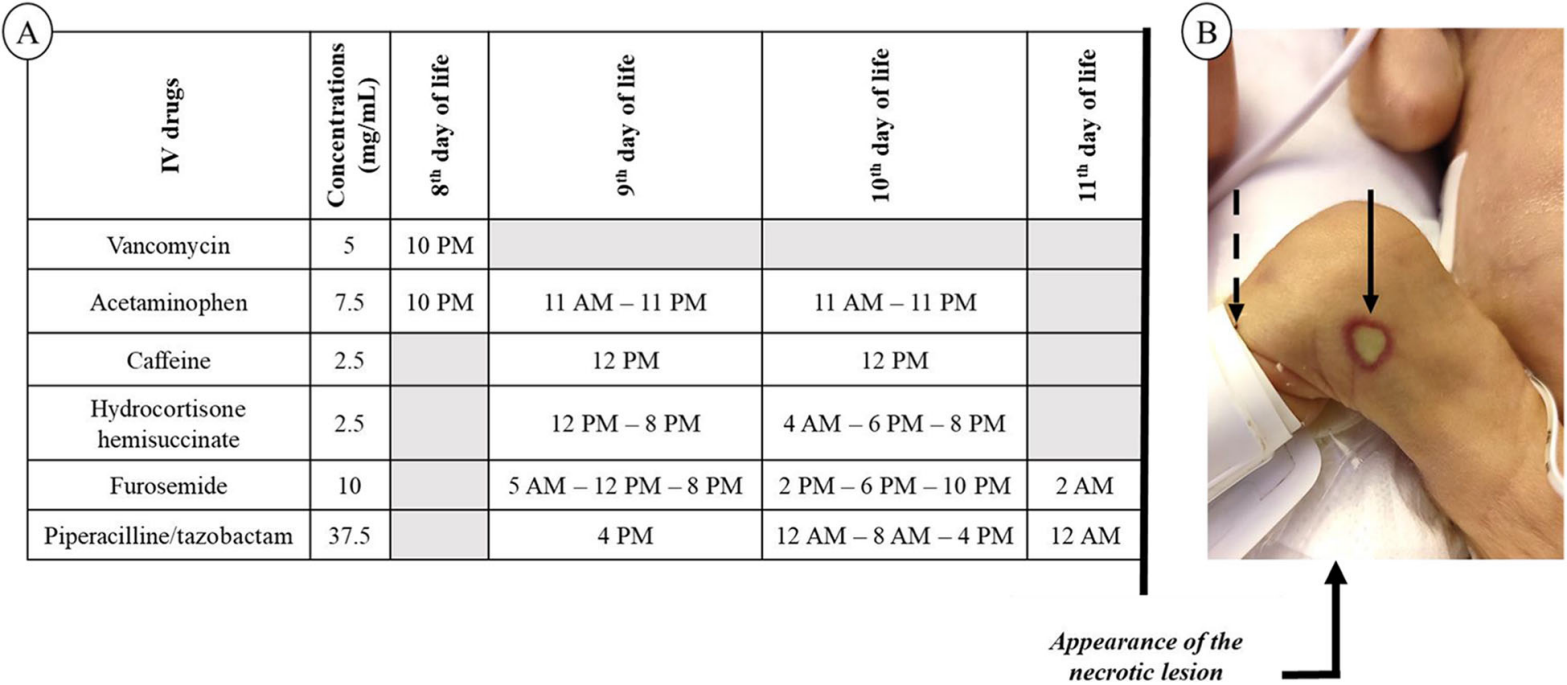
A. Daily chronology of injections on the peripheral venous catheter. B. Skin necrotic lesion related to vancomycin infusion appeared in the flow area of the vein of the right leg. The dotted arrow points out the injection site on the left and the plain arrow points out the skin necrosis area on the right. Legend: IV, intravenous

The use of continuous high-risk medications (insulin, dopamine and prostin) required the insertion of a peripheral venous catheter, dedicated to intermittent injections. The following drugs were infused through the catheter: piperacillin-tazobactam, furosemide, caffeine citrate, acetaminophen, hydrocortisone succinate and vancomycin. Vancomycin was administered two hours after catheterization via a one-hour long infusion. Forty-eight hours later, a skin necrotic lesion appeared progressively one inch above the injection-site (shown in Fig. 1).

The intravenous line was immediately removed. A sterile gauze dressing was applied on the necrotic zone until its total regression. The case was declared to the Lille Regional Pharmacovigilance Centre (number LL20201014).

## Discussion/conclusion

Among the injectable medications administered via a peripheral venous catheter, vancomycin has been reported to irritate the vascular wall, due to its acid (2.8–4.5) pH. The fact that a similar delay between administration of vancomycin and development of a skin lesion has been reported previously in an adult patient reinforced our impression that the skin lesion was related to vancomycin infusion [4]. The vancomycin concentration of 5 mg/mL that was peripherally administered in our case is widely used in infants or neonates and had not yet been reported to be harmful. However, an in vitro study showed that concentrations of vancomycin under 2.5 mg/mL in contact with endothelial cells had no impact on cell viability after 72 h of treatment, with a probability of survival above 80%. Their results highlighted that the endothelial toxicity increases with the concentration of vancomycin above that 2.5 mg/mL-limit, so that 5 mg/mL vancomycin induces a loss of viability of 50% of the initial pool of endothelial cells within 24 h. Vancomycin may therefore damage endothelial cell significantly from a 2.5 mg/mL concentration [5].

It is of current knowledge that vancomycin is preferentially given continuously using a central catheter. In NICU, the central venous catheter is often dedicated to the administration of continuous low-rate medications and the peripheral venous catheter to the administration of intermittent injections. Concerning the injection of vancomycin, the loading dose is preferentially given on central catheter. However, it may be given via peripheral venous catheter when the use of numerous continuous medications and their potential incompatibilities with vancomycin require the use of independent peripheral lines. Otherwise, a peripheral administration of the loading dose of vancomycin will also be preferred if the only available central venous line is used to administer continuous injectable medications with narrow therapeutic ranges to the patient (i.e. insulin, norepinephrine, heparin injection) because of the high risk of increasing harmfully the administration rate of these latter drugs.

Critically ill newborns have multiple risk factors for adverse outcomes including an immature immune system and compromised skin integrity. Their inflammatory and hypercatabolic state influence the high susceptibility of this population to vancomycin endothelial toxicity. Endothelial toxicity of vancomycin must be considered as skin necrosis can be an entry point to a bacteremia and thus worsen the outcome of the vulnerable preterm newborn infants.

The combination of irritant intravenous drugs administered via a peripheral venous catheter, i.e. furosemide and caffeine in our case, may have enhanced the cumulative endothelial toxicity. A recent article tested the toxicity on endothelial cells viability when vancomycin was combined with piperacillin-tazobactam, revealing no excess cell death compared with the cell death rate from vancomycin alone [6]. However, there is no evidence that vancomycin was here the only irritant drug related to the skin lesion. The assumption of the potentiation of the irritant effect by the other intravenous agents should be considered.

Finally, evidence suggests that presence of particles in infusion fluid was a major cause of chemical phlebitis [7], supporting that use of in-line intravenous filters could reduce infusion particles [8].

To prevent any damaging effect, the administration of vancomycin at a concentration lower than 2.5 mg/mL should be recommended when a central venous catheter is not available for the administration of injectable vancomycin. The combination of irritant injectable drugs administered via a peripheral venous catheter should be avoided even when the catheter is flushed with normal saline after each infusion, to limit the cumulative endothelial toxicity at the venous access point. The use of in-line intravenous filter must be considered for each antibiotic infusion in critically ill infants.

## Data Availability

Not applicable.

## Abbreviations

NICU: Neonatal intensive care unit
BiPAP: Biphasic Positive air pressure
